# Antidepressant Use and Risk of Manic Episodes in Children and Adolescents With Unipolar Depression

**DOI:** 10.1001/jamapsychiatry.2023.3555

**Published:** 2023-09-27

**Authors:** Suvi Virtanen, Tyra Lagerberg, Christine Takami Lageborn, Ralf Kuja-Halkola, Isabell Brikell, Anthony A. Matthews, Paul Lichtenstein, Brian M. D’Onofrio, Mikael Landén, Zheng Chang

**Affiliations:** 1 Department of Medical Epidemiology and Biostatistics, Karolinska Institutet, Stockholm, Sweden; 2 Department of Psychiatry, Warneford Hospital, University of Oxford, Oxford, United Kingdom; 3 Unit of Epidemiology, Institute of Environmental Medicine, Karolinska Institute, Stockholm, Sweden; 4 Department of Psychological and Brain Sciences, Indiana University, Bloomington; 5 Institute of Neuroscience and Physiology, Sahlgrenska Academy, Gothenburg University, Gothenburg, Sweden

## Abstract

**Importance:**

Antidepressants are increasingly prescribed to pediatric patients with unipolar depression, but little is known about the risk of treatment-emergent mania. Previous research suggests pediatric patients may be particularly vulnerable to this adverse outcome.

**Objective:**

To estimate whether pediatric patients treated with antidepressants have an increased incidence of mania/hypomania compared with patients not treated with antidepressants and to identify patient characteristics associated with the risk of mania/hypomania.

**Design, Setting, and Participants:**

In a cohort study applying the target trial emulation framework, nationwide inpatient and outpatient care in Sweden from July 1, 2006, to December 31, 2019, was evaluated. Follow-up was conducted for 12 and 52 weeks after treatment initiation, with administrative follow-up ending December 31, 2020. Data were analyzed between May 1, 2022, and June 28, 2023. Individuals aged 4 to 17 years with a diagnosis of depression, but without a prior diagnosis of mania/hypomania, bipolar disorder, or psychosis or treatment with mood stabilizer (lithium, valproate, or carbamazepine), prescriptions were included.

**Exposures:**

The treatment group included patients who initiated any antidepressant medication within 90 days of diagnosis. The control group included patients who did not initiate antidepressants within 90 days.

**Main Outcomes and Measures:**

Diagnosis of mania/hypomania or initiation of mood stabilizer therapy. Incidences were estimated with Kaplan-Meier estimator, and inverse probability of treatment weighting was used to adjust for group differences at baseline.

**Results:**

The cohort included 43 677 patients (28 885 [66%] girls); 24 573 in the treatment group and 19 104 in the control group. The median age was 15 (IQR, 14-16) years. The outcome occurred in 96 individuals by 12 weeks and in 291 by 52 weeks. The cumulative incidence of mania was 0.26% (95% CI, 0.19%-0.33%) in the treatment group and 0.20% (95% CI, 0.13%-0.27%) in the control group at 12 weeks, with a risk difference of 0.06% (95% CI, −0.04% to 0.16%). At 52 weeks, the cumulative incidence was 0.79% (95% CI, 0.68%-0.91%) in the treatment group and 0.52% (95% CI, 0.40%-0.63%) in the control group (risk difference, 0.28%; 95% CI, 0.12%-0.44%). Hospitalizations, parental bipolar disorder, and use of antipsychotics and antiepileptics were the most important predictors of mania/hypomania by 12 weeks.

**Conclusion:**

This cohort study found no evidence of treatment-emergent mania/hypomania by 12 weeks in children and adolescents. This corresponds to the time frame for antidepressants to exert their psychotropic effect. A small risk difference was found only with longer follow-up. Certain patient characteristics were associated with mania/hypomania, which warrants clinical attention.

## Key Points

**Question:**

Do pediatric patients diagnosed with unipolar depression who receive antidepressant treatment have an increased incidence of mania/hypomania compared with patients not treated with antidepressants?

**Findings:**

In this cohort study including 43 677 children and adolescents diagnosed with unipolar depression, during a 12-week follow-up, patients treated with antidepressants did not have an increased incidence of mania/hypomania compared with patients who did not initiate antidepressant treatment. Hospitalizations, parental bipolar disorder, and use of antipsychotics and antiepileptics were the most important predictors of mania/hypomania by 12 weeks.

**Meaning:**

This study found no evidence of treatment-emergent mania/hypomania in children and adolescents with unipolar depression.

## Teaser

This cohort study examines the risks for and incidence of mania episodes after 12 and 52 weeks in children and adolescents with unipolar depression who are prescribed antidepressants.

## Introduction

Depression is a prevalent psychiatric disorder among children and adolescents,^1,2,3,4^ for which antidepressants, such as selective serotonin reuptake inhibitors (SSRIs), are the recommended first-line pharmacologic treatment.^5^ Some antidepressants, especially tricyclic antidepressants, may induce manic symptoms in a subset of patients.^6,7,8^ While considered a safer option, similar concerns have been raised for SSRIs and serotonin/norepinephrine reuptake inhibitors.^7,9,10^ Treatment-emergent switch, ie, the transition from depression into mania shortly after the initiation of antidepressant treatment, is estimated to occur in 3% to 10% of adult patients with bipolar disorder.^11,12^ However, it remains unclear whether antidepressants can induce mania in patients with unipolar depression. Randomized clinical trials (RCTs) showed no clinically meaningful risk difference between adults with unipolar depression receiving SSRIs vs placebo or tricyclic antidepressants vs placebo,^12^ whereas observational studies reported a clearly increased risk.^7,9,10,13^

A register-based study found a particularly increased risk of manic conversion in children medicated with SSRIs.^14^ This finding, along with reports of other adverse medication-related outcomes, such as aggression^15,16,17,18^ and suicidality,^15,19,20^ have raised concerns about the safety of antidepressants.^21,22,23^ This is particularly pressing given that antidepressants are increasingly prescribed to children and adolescents.^24,25,26^ Some studies suggest that the developing brain might be especially vulnerable to adverse drug reactions,^23,27,28,29^ although the mechanisms remain unknown.

It is critical to understand potential risks related to antidepressants in pediatric patients, but rare outcomes, such as incident mania, are difficult to investigate in RCTs. Randomized clinical trials often have insufficient sample sizes and strict inclusion criteria, which correspond poorly with the characteristics of the population actually receiving the medication in clinical practice.^7,8,21,22^ For instance, most pediatric antidepressant trials excluded individuals at risk of developing mania, such as those with a family history of bipolar disorder or who received inpatient treatment. Mania occurred in 0 to 4 patients and was not evaluated or was not reported in trials with a sample size from 96 to 376 individuals.^21,22^ Electronic health record data circumvent these limitations, but observational studies using such data may be confounded if not designed and analyzed appropriately.^9,10,14,21^ Furthermore, if the goal is to investigate whether antidepressant initiation is associated with the incidence of mania, it is important to specify a clinically meaningful follow-up time. This means that observational studies should specify the time frame after antidepressant initiation in which newly emergent mania could be linked with antidepressants and not to the natural course of bipolar disorder misdiagnosed as unipolar. The International Society for Bipolar Disorders Task Force suggests 8 weeks, which is analogous to the customary time frame in which antidepressant efficacy is determined.^30,31,32^ Most medication-induced adverse reactions also occur within the first months of treatment.^33,34^ Prior observational studies followed up patients over several years,^13,14^ making it unlikely that outcome differences can be attributed solely to medication initiation.

Identifying individuals with an increased risk of developing mania may improve outcomes by providing information on which patients should be monitored closely during treatment. In addition to antidepressants, potential predictors of switch to mania include female sex, family history of mood disorders, psychotic features, emotional/behavioral dysregulation, hospitalization, and younger age.^35^ However, studies focusing on children and adolescents are scarce, and most prior work relied on small clinical samples.^35^

This study investigated the risk of incident mania/hypomania in pediatric patients with unipolar depression using nationwide register data from Sweden. We estimated the risk at 12 and 52 weeks of follow-up. We postulated that if antidepressants were to induce mania/hypomania, differences between the groups would emerge by 12 weeks. We used a time frame suggested by the International Society for Bipolar Disorders, adding 4 weeks to the customary 8 weeks to account for a potentially slower titration schedule in pediatric patients.^36^ It is less likely that the risk difference can be attributed to medication initiation if it emerges only in the longer follow-up period. Additionally, we investigated which patient characteristics predicted mania/hypomania in the 12-week follow-up.

## Methods

### Data Sources

In this cohort study, we used Swedish nationwide administrative registers linked with the unique personal identity number assigned at birth or upon immigration.^37,38^ Diagnoses were retrieved from the National Patient Register, which covers diagnoses from inpatient and outpatient care.^39^ Information concerning prescribed drugs was obtained from the Prescribed Drug Register, which contains data on all dispensed prescribed drugs since July 2005.^40^ Because the cohort was composed of children and adolescents, socioeconomic covariates were based on their parents’ information. Cohort members were linked to their parents via the Multi-Generation Register.^41^ We retrieved information on parental income and educational level from Statistics Sweden’s registers. The study was approved by the Swedish Ethical Review Authority. Informed consent requirement was waived by the Swedish Ethical Review Authority because the study was register based, and data were pseudonymized. This study followed the Strengthening the Reporting of Observational Studies in Epidemiology (STROBE) reporting guideline.

### Study Cohort

We identified children and adolescents, aged 4 to 17 years, diagnosed with unipolar depression (*International Statistical Classification of Diseases and Related Health Problems, Tenth Revision* [*ICD-10*] codes F32 to F33, excluding F33.4) between July 1, 2006, and December 31, 2019. We required a washout period without use of any antidepressant medication (*Anatomical Therapeutic Classification* [*ATC*] code N06A) for at least 1 year preceding the depression diagnosis. Since the study focused on incident mania/hypomania, we excluded individuals with a prior diagnosis of mania (*ICD-10* code F30), bipolar disorder (*ICD-10* code F31), and psychosis (*ICD-10* code F2) or prescriptions for lithium (*ATC* code N05AN), valproate (*ATC* code N03AG01), or carbamazepine (*ATC* code N03AF01) recorded any time before the depression diagnosis. We required that the prescribing indication was for mood-stabilizing purpose for the latter 2 medications. We selected the first diagnosis registered during the study period as the index diagnosis for eligible participants. Data were analyzed between May 1, 2022, and June 28, 2023.

### Exposure

The treatment group consisted of patients who initiated any antidepressant medication within 90 days of diagnosis. The control group included patients who did not initiate antidepressant treatment within 90 days of diagnosis.

### Outcome

The outcome was defined as any of the following: diagnosis of mania (*ICD-10* code F30 [F30.0 hypomania]) or bipolar disorder (*ICD-10* codes F31.0 [hypomania], F31.1, F31.2, and F31.6), or the initiation of lithium, valproate, or carbamazepine within 12 and 52 weeks of follow-up start.

### Covariates

Several clinical and sociodemographic covariates were measured at baseline, including sex, age, year of diagnosis, source of diagnosis (inpatient vs outpatient), other diagnoses recorded any time before the follow-up start (anxiety disorders, attention deficit/hyperactivity disorder, drug use disorder, alcohol use disorder, personality disorders, conduct disorders, developmental disorders, autism spectrum disorder, suicide attempt/self-harm, and poisoning/overdose), other medication use within 4 months before the follow-up start (antipsychotics, hypnotics/sedatives, antiepileptics, stimulants, and opioids), number of mental health hospitalizations recorded any time before the follow-up start (as a proxy for the severity of mental health problems), parental income, parental educational level, and parental bipolar and depressive disorders. A detailed description of covariates is available in eTable 1 in Supplement 1. Information was missing for 1 or more covariates in a subset of individuals (n = 2197). If information was missing, the variable was coded as unknown and modeled as a separate response category.

### Statistical Analysis

We applied the target trial emulation framework to guide the study design and analysis^42^ (eTable 2 in Supplement 1). Briefly, the first step for conducting a target trial emulation is articulating the causal question in the form of the protocol of a hypothetical randomized trial that would provide the answer. The protocol must specify certain key elements that define the causal estimands (eligibility criteria, treatment strategies, treatment assignment, start and end of follow-up, outcomes, and causal contrasts). The randomized trial described in the protocol becomes the target study, which is then emulated as closely as possible within the constraints of the observational data available. We provide the protocol in eTable 2 in Supplement 1. For the treatment group, follow-up started at the date of the first medication dispensation. The number of days from diagnosis to the first dispensation was assessed for participants who began treatment. For each person who did not initiate therapy, follow-up start was matched at random from this set and assigned to them (eFigure 1 in Supplement 1), which ensures the same distribution of time between diagnosis and start of follow-up in both groups, therefore accounting for immortal time bias.^43^ We estimated intention-to-treat (ITT) and per-protocol effect. In ITT, switching from antidepressant treatment to no treatment after the 90-day period and vice versa were not accounted for. In the per-protocol analysis, participants were censored when they switched from treatment to no treatment or vice versa. Details of this analysis are available in the eMethods in Supplement 1.

Follow-up continued until 12 weeks to investigate short-term outcomes. A time frame of 8 weeks is consistent with efficacy trials and suggested as the maximum time in which newly emergent mania may be considered antidepressant induced.^30,31^ We added 4 more weeks to account for a potentially slower titration schedule in pediatric patients^36^ as well as potential delay between medication dispensation and the time the patient began the medication. As a separate analysis, we extended follow-up to 52 weeks. Follow-up continued until emigration, death, outcome, censoring for psychosis hospitalization, administrative censoring at 12 and 52 weeks, administrative censoring on December 31, 2020, or censoring for treatment switch in the per-protocol analysis, whichever occurred first. We examined potential sex differences by estimating the models separately for boys and girls.

We used inverse probability of treatment weighting to adjust for differences between groups at baseline. The baseline covariates were used to estimate a propensity score, which is the probability of treatment conditional on covariates. We used stabilization and truncating to avoid extreme weights. For stabilized weights, the numerator of the weight is the probability of receiving the observed treatment. The denominator of the weight is the probability that given baseline confounders, an individual receives their observed treatment (ie, the numerator is the propensity score).^44^ The weights were then truncated at the 99th percentile. We used the resulting weights in the estimation of Kalbfleisch-Prentice cumulative incidence curves and Cox proportional hazards regression models. Confidence intervals were calculated using the infinitesimal jackknife variance estimator.

In sensitivity analyses, we assessed the robustness of the main results by using different cohort, exposure, outcome, and follow-up definitions. We (1) excluded individuals with missing information in any covariate; (2) excluded individuals who were dispensed antipsychotics or antiepileptics within 4 months before the start of follow-up; (3) extended the grace period to 120 days after diagnosis when defining the treatment group; (4) considered outcome to have occurred only if the individual had at least 2 diagnoses or 1 diagnosis and 1 medication dispensation; (5) extended the outcome to include olanzapine (*ATC* code N05AH03); included only prescriptions with an indication for a mood-stabilizing purpose (olanzapine was selected because, in addition to medications in the primary outcome, it is among the recommended treatment options for mania in pediatric patients in Sweden^45^); (6) extended the follow-up to 18 weeks; (7) censored individuals if they were hospitalized for any mental health reason (excluding the outcome); (8) conducted cloning-censoring-weighting analysis, in which follow-up starts at the index diagnosis^46^ to test whether our main approach was robust to immortal time bias (details in the eMethods in Supplement 1); and (9) used alternative ways to define treatment periods in per-protocol analyses (eMethods in Supplement 1).

We investigated which patient characteristics (covariates specified earlier) were associated with mania/hypomania in the 12-week follow-up. We used multivariable Cox proportional hazards regression to estimate a hazard ratio for each predictor. Some predictor variables did not have a sufficient number of observations to estimate a separate coefficient. We therefore recoded or combined a subset of them (details in the eMethods in Supplement 1). Predictive performance of the model was assessed with the concordance index. In the second step, we included the initiation of antidepressants to the model to test whether including it added incremental value in predicting mania/hypomania.

## Results

The cohort included 43 677 patients (28 885 [66%] girls; 14 792 [34%] boys) of whom 24 573 (56%) initiated antidepressant treatment within 90 days of diagnosis; median age was 15 (IQR, 14-16) years. More than 95% of individuals who initiated treatment received SSRIs, with fluoxetine and sertraline being the most common types (eTable 3 in Supplement 1). Time from diagnosis to antidepressant initiation was short (mean, 10 days; median, 1 day [IQR, 0-11 days]), and 86% initiated antidepressants within 4 weeks. The estimated treatment periods lasted a mean (SD) of 398 (441) days (median, 250 [IQR, 113-513] days). A minority (11%) of the participants were dispensed only 1 antidepressant prescription. The outcome occurred in 96 individuals by 12 weeks and in 291 by 52 weeks.

Table 1 reports the nonweighted descriptive statistics of the cohort. The inverse probability of treatment-weighted descriptive statistics are available in eTable 4 in Supplement 1, showing that weighting produced excellent balance between groups in all covariates. A standardized mean difference smaller than 0.1 suggests a good balance between groups. The stabilized treatment weights had a mean of 0.995 (IQR, 0.751-1.113). A mean of 1 is considered to indicate no clear evidence of misspecification of the propensity model.^47^

**Table 1.** Nonweighted Cohort Characteristics^a^

Characteristic	No. (%)		SMD (95% CI)
	Control (n = 19 104)	Treatment (n = 24 573)	
Sex			
Male	6827 (35.7)	7965 (32.4)	0.070 (0.051 to 0.089)
Female	12 277 (64.3)	16 608 (67.6)	
Age, mean (SD), y	14.66 (2.06)	15.12 (1.72)	0.240 (0.223 to 0.260)
Parental educational level			
Primary school	926 (4.8)	855 (3.5)	0.150 (0.130 to 0.168)
High school	8081 (42.3)	10 433 (42.5)	
University	9197 (48.1)	12 698 (51.7)	
Unknown	900 (4.7)	587 (2.4)	
Family income percentile			
<20	1364 (7.1)	1365 (5.6)	0.153 (0.134 to 0.172)
20-80	12 520 (65.5)	16 152 (65.7)	
>80	4241 (22.2)	6367 (25.9)	
Unknown	979 (5.1)	689 (2.8)	
Diagnosis year, mean (SD)	2013.89 (3.71)	2014.06 (3.70)	0.047 (0.029 to 0.066)
Source of diagnosis			
Outpatient	17 422 (91.2)	22 819 (92.9)	0.071 (0.052 to 0.089)
Inpatient	1432 (7.5)	1566 (6.4)	
Unknown	250 (1.3)	188 (0.8)	
Parental bipolar disorder			
No	17 562 (91.9)	23 021 (93.7)	0.104 (0.085 to 0.123)
Yes	804 (4.2)	1035 (4.2)	
Unknown	738 (3.9)	517 (2.1)	
Parental depression			
No	15 742 (82.4)	20 689 (84.2)	0.104 (0.085 to 0.123)
Yes	2623 (13.7)	3368 (13.7)	
Unknown	739 (3.9)	516 (2.1)	
Prior hospitalizations			
0	16 655 (87.2)	20 801 (84.6)	0.078 (0.059 to 0.097)
1	1862 (9.7)	2887 (11.7)	
2 or 3	475 (2.5)	767 (3.1)	
≥4	112 (0.6)	118 (0.5)	
Diagnoses			
Anxiety disorder	4898 (25.6)	8035 (32.7)	0.156 (0.137 to 0.175)
Eating disorder	1361 (7.1)	1962 (8.0)	0.033 (0.014 to 0.051)
Personality disorder	240 (1.3)	320 (1.3)	0.004 (−0.015 to 0.023)
ADHD	2908 (15.2)	3317 (13.5)	0.049 (0.030 to 0.068)
Developmental disorder	1683 (8.8)	2125 (8.6)	0.006 (−0.013 to 0.025)
Autism spectrum disorder	795 (4.2)	1626 (6.6)	0.109 (0.090 to 0.128)
Conduct disorder	549 (2.9)	574 (2.3)	0.034 (0.015 to 0.053)
Alcohol use disorder	431 (2.3)	467 (1.9)	0.025 (0.006 to 0.044)
Drug use disorder	444 (2.3)	410 (1.7)	0.047 (0.028 to 0.066)
Poisoning by drugs	1061 (5.6)	1066 (4.3)	0.056 (0.037 to 0.075)
Alcohol poisoning	33 (0.2)	32 (0.1)	0.011 (0.037 to 0.075)
Suicidal behavior	1323 (6.9)	1418 (5.8)	0.047 (0.028 to 0.066)
Medications			
Antipsychotics	294 (1.5)	631 (2.6)	0.073 (0.054 to 0.092)
Hypnotics/sedatives	4365 (22.8)	11 538 (47.0)	0.523 (0.503 to 0.542)
Benzodiazepines	40 (0.2)	164 (0.7)	0.069 (0.050 to 0.088)
Antiepileptics	249 (1.3)	198 (0.8)	0.049 (0.030 to 0.068)
Opioids	171 (0.9)	241 (1.0)	0.009 (−0.010 to 0.028)
Stimulants	1719 (9.0)	2017 (8.2)	0.028 (0.009 to 0.047)

Abbreviations: ADHD, attention deficit/hyperactivity disorder; SMD, absolute standardized mean difference.

^a^ The inverse probability of treatment-weighted cohort characteristics are available in eTable 4 in Supplement 1.

In the ITT analysis at 12 weeks, the estimated cumulative incidence of mania/hypomania was 0.26% (95% CI, 0.19%-0.33%) in the treatment group and 0.20% (95% CI, 0.13%-0.27%) in the control group, corresponding to a risk difference of 0.06% (95% CI, −0.04% to 0.16%) and a hazard ratio of 1.29 (95% CI, 0.83-2.03) (Table 2). The weighted risk curves are presented in the Figure. By 52 weeks, the cumulative incidence was 0.79% (95% CI, 0.68%-0.91%) in the treatment group and 0.52% (95% CI, 0.40%-0.63%) in the control group, with a risk difference of 0.28% (95% CI, 0.12%-0.44%) and a hazard ratio of 1.54 (95% CI, 1.18-2.00). The weighted risk curves are presented in eFigure 2 in Supplement 1. Nonweighted results show that weighting produced slightly attenuated associations (Table 2; eFigure 2 and eFigure 3 in Supplement 1). Boys in the treatment group appeared to have an increased risk of mania/hypomania relative to controls compared with the relative risk in girls (eTable 5 in Supplement 1). However, 95% CIs were highly overlapping. Per-protocol results were similar to the ITT estimates at 12 weeks, possibly showing attenuation at 52 weeks (Table 2).

**Table 2.** Main Analyses: Cumulative Incidence and Relative Risk of Mania/Hypomania at 12 and 52 Weeks

Outcome	No. of patients	No. of events	With IPT weighting (95% CI)			Without IPT weighting (95% CI)		
			Cumulative incidence, %	Risk difference, %	Hazard ratio^a^	Cumulative incidence, %	Risk difference, %e	Hazard ratio^a^
**Intention to treat**								
12 wk^b^								
Control	19 104	36	0.20 (0.13 to 0.27)	0.06 (−0.04 to 0.16)	1.29 (0.83 to 2.03)	0.19 (0.13 to 0.25)	0.06 (−0.03 to 0.14)	1.30 (0.86 to 1.96)
Treatment	24 573	60	0.26 (0.19 to 0.33)			0.24 (0.18 to 0.31)		
52 wk^c^								
Control	19 104	95	0.52 (0.40 to 0.63)	0.28 (0.12 to 0.44)	1.54 (1.18 to 2.00)	0.50 (0.39 to 0.60)	0.30 (0.15 to 0.45)	1.61 (1.25 to 2.05)
Treatment	24 573	196	0.79 (0.68 to 0.91)			0.80 (0.69 to 0.91)		
**Per protocol**								
12 wk								
Control	19 104	36	0.20 (0.08 to 0.31)	0.02 (−0.13 to 0.17)	1.11 (0.53 to 2.32)	0.19 (0.12 to 0.26)	0.02 (−0.06 to 0.11)	1.12 (0.71 to 0.77)
Treatment	24 573	49	0.22 (0.11 to 0.33)			0.21 (0.16 to 0.27)		
52 wk								
Control	19 104	78	0.48 (0.28 to 0.68)	0.15 (−0.11 to 0.41)	1.31 (0.79 to 2.16)	0.44 (0.35 to 0.54)	0.21 (0.06 to 0.35)	1.46 (1.11 to 1.93)
Treatment	24 573	116	0.63 (0.44 to 0.82)			0.65 (0.53 to 0.76)		

Abbreviation: IPT, inverse probability of treatment.

^a^ Per-protocol analyses estimated risk ratios instead of hazard ratios because a discrete-time hazards model (a pooled logistic model) was used.

^b^ A total of 56% (n = 54) of the reported outcomes were based on diagnoses.

^c^ A total of 49% (n = 142) of the reported outcomes were based on diagnoses.

**Figure.**
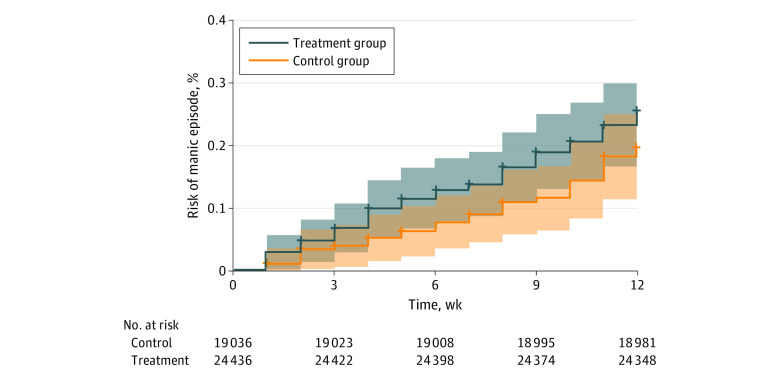
Estimated Cumulative Incidence of Mania/Hypomania During 12 Weeks of Follow-Up Data were weighted by the treatment propensity weights. Shaded areas represent 95% CIs; the gray shaded area indicates overlapping 95% CIs.

In the sensitivity analyses with different cohort, exposure, follow-up, and outcome definitions, the estimates remained similar to the main analyses (Table 3; nonweighted data given in eTable 6 in Supplement 1). Table 4 reports the association of baseline patient characteristics with the risk of mania/hypomania by 12 weeks. Hospitalization was associated with a 2.1-fold increased risk of mania/hypomania by 12 weeks, inpatient as the source of diagnosis with a 2.3-fold increased risk, parental bipolar disorder with a 4.1-fold increased risk, use of antipsychotics with a 4.4-fold increased risk, and use of antiepileptics with a 7.0-fold increased risk. The concordance index of the model was 0.77. Of all outcomes, 58% occurred in patients who had at least 1 of the top 5 predictors (8436 [20%] of the cohort). When antidepressant treatment was added to the model, it was not associated with the outcome, nor did the concordance index of the model change after its inclusion.

**Table 3.** Sensitivity Analyses: Cumulative Incidence and Relative Risk of Mania/Hypomania at 12 Weeks^a^

Outcome	Cumulative incidence, % (95% CI)		Risk difference (95% CI)	Hazard ratio
	Control	Treatment		
Complete cases^b^	0.20 (0.14 to 0.30)	0.23 (0.17 to 0.30)	0.03 (−0.07 to 0.13)	1.15 (0.72 to 1.83)
Excluding certain medication users^c^	0.16 (0.10 to 0.22)	0.20 (0.14 to 0.26)	0.04 (−0.04 to 0.13)	1.28 (0.78 to 2.09)
Extended grace period^d^	0.21 (0.15 to 0.28)	0.26 (0.19 to 0.33)	0.05 (−0.06 to 0.15)	1.21 (0.78 to 1.89)
Restricted outcome definition^e^	0.08 (0.04 to 0.11)	0.06 (0.03 to 0.09)	−0.02 (−0.07 to 0.04)	0.80 (0.36 to 1.74)
Extended outcome definition^f^	0.21 (0.14 to 0.28)	0.27 (0.20 to 0.34)	0.06 (−0.04 to 0.16)	1.29 (0.83 to 2.00)
Extended follow-up length^g^	0.25 (0.17 to 0.33)	0.35 (0.27 to 0.43)	0.10 (−0.01 to 0.21)	1.39 (0.95 to 2.04)
Censoring for any hospitalization^h^	0.18 (0.11 to 0.24)	0.24 (0.17 to 0.30)	0.06 (−0.04 to 0.15)	1.34 (0.83 to 2.15)
Cloning-censoring weighting, ITT^i^	0.22 (0.16 to 0.30)	0.22 (0.18 to 0.27)	0.00 (−0.09 to 0.08)	1.01 (0.68 to 1.48)
Cloning-censoring-weighting, PP^i^	0.22 (0.16 to 0.31)	0.21 (0.17 to 0.26)	−0.01 (−0.08 to 0.10)	0.93 (0.62 to 1.39)
60-d Gap treatment periods, PP^j^	0.20 (0.08 to 0.31)	0.22 (0.11 to 0.32)	0.02 (−0.14 to 0.17)	1.09 (0.51 to 2.32)
1 Pill/d treatment periods, PP^k^	0.20 (0.08 to 0.32)	0.20 (0.09 to 0.32)	0.00 (−0.15 to 0.16)	1.01 (0.49 to 2.10)

Abbreviations: ITT, intention to treat; PP, per protocol.

^a^ Estimates are from the ITT approach unless stated otherwise.

^b^ Individuals with missing information in any covariate were excluded (cohort n = 41 480).

^c^ Individuals who were dispensed any antipsychotic or antiepileptic medications within 4 months before initiation of follow-up were excluded (cohort n = 42 346).

^d^ The grace period was extended to 120 days after diagnosis in defining the treatment group.

^e^ Outcomes were restricted to those that occurred only in individuals who had at least 2 diagnoses or 1 diagnosis and 1 medication dispensation.

^f^ Outcome was extended to include dispensations for olanzapine (with indication for mood-stabilizing purpose).

^g^ Follow-up length was extended from 12 to 18 weeks.

^h^ Individuals were censored if they were hospitalized for any mental health reason (excluding the outcome).

^i^ Cloning-censoring weighting approach is described in detail in the eMethods in Supplement 1. Follow-up began from the time of the index diagnosis.

^j^ When defining antidepressant treatment periods, it was assumed that 2 dispensations falling within 60 days of each other belonged to the same treatment period. See the eMethods in Supplement 1 for details of this analysis.

^k^ Antidepressant treatment period lengths were estimated from the total quantity of pills dispensed and assuming that patients receive 1 pill per day. See the eMethods in Supplement 1 for details of this analysis.

**Table 4.** Patient Characteristics Associated With Mania/Hypomania in the 12-Week Follow-Up, Ranked According to Effect Size

Predictor	No. (%)^a^	HR (95% CI)
Use of antiepileptics	410 (1.0)	7.01 (3.31-14.84)^b^
Use of antipsychotics	845 (2.0)	4.42 (2.16-9.06)^b^
Parental bipolar disorder	1810 (4.4)	4.09 (2.24-7.44)^b^
Inpatient as source of diagnosis	2790 (6.7)	2.27 (1.13-4.58)^b^
Any prior hospitalization	5709 (13.8)	2.12 (1.08-4.17)^b^
Developmental disorder	3618 (8.7)	1.47 (0.79-2.73)
Use of stimulants	3542 (8.5)	1.39 (0.56-3.41)
Parental tertiary-level education	21 564 (52.0)	1.31 (0.84-2.02)
Personality or conduct disorder	1524 (3.7)	1.21 (0.50-2.89)
Alcohol or drug poisoning	2018 (4.9)	1.13 (0.35-3.61)
Age	NA^c^	1.08 (0.96-1.22)
ADHD	5915 (14.3)	1.07 (0.47-2.40)
Anxiety disorder	11 984 (28.9)	1.03 (0.65-1.65)
Parental depression	5873 (14.2)	1.03 (0.59-1.80)
Parental income >80th percentile	10 496 (25.3)	1.02 (0.62-1.70)
Use of sedatives	15 297 (36.9)	1.01 (0.65-1.59)
Year of diagnosis	NA^c^	0.93 (0.87-0.98)^b^
Female sex	27 784 (67.0)	0.87 (0.56-1.35)
Autism spectrum disorder	2257 (5.4)	0.45 (0.16-1.30)
Suicidal behavior	2528 (6.1)	0.34 (0.10-1.19)
Eating disorder	3197 (7.7)	0.32 (0.10-1.06)
Substance use disorder	1512 (3.6)	0.26 (0.06-1.10)

Abbreviations: ADHD, attention deficit/hyperactivity disorder; HR, hazard ratio; NA, not applicable.

^a^ Percentage represents the proportion of individuals with the predictor.

^b^ Statistically significant coefficient.

^c^ Continuous variable for which the percentage cannot be calculated.

## Discussion

This study investigated the risk of incident mania/hypomania in a cohort of pediatric patients with unipolar depression in Sweden. We found no evidence to suggest that antidepressants induce mania/hypomania in this patient group. The risk was similar in treatment and control groups by 12 weeks, which is the time window during which treatment-emergent mania is expected to occur. We found a small risk difference only in the longer follow-up analysis, suggesting that the risk increase may be attributable to factors other than the medication. We identified several patient characteristics that predicted mania/hypomania.

Our results contrast with an earlier register-based study reporting a substantially increased risk of mania in pediatric patients with unipolar depression treated with antidepressants.^14^ Differences in the results are likely explained by differences in study design. By using the target trial emulation framework, we aimed to minimize common biases in observational cohort studies.^42^ The most important differences, however, relate to the length of follow-up. The prior study followed up patients up to 5 years and excluded outcomes within the first 2 months. We used a shorter follow-up of 12 weeks consistent with the International Society for Bipolar Disorders Task Force consensus for defining treatment-emergent manic switch^31,32^ and did not exclude early outcomes. Conversion to mania long after treatment initiation may rather reflect selection bias, as patients with underlying bipolar disorder are also more likely to be prescribed antidepressants, and mania would have occurred eventually regardless of medication. We also observed a modest risk difference of 0.28% in the 52-week follow-up, which was further attenuated in the per-protocol analysis. The increased risk in this longer follow-up was likely produced by the combination of unadjusted time-varying confounding and the mentioned selection mechanism.

However, our findings are in line with RCTs.^12,21,22^ Similarly to RCTs, our cohort study found incident mania to be rare in the clinical practice setting. Furthermore, RCTs have found no clear evidence of antidepressants increasing the risk of mania compared with placebo.^21,22^ There have been concerns of clinical trials having inadequate data on adverse outcomes and for excluding individuals with the highest risk of developing mania.^21,22^ Our data offer complementary information to RCTs from a large cohort of patients treated in actual clinical practice. In addition, our findings converge with a recent study in adults with bipolar disorder, which found no increased risk of new-onset mania following antidepressant initiation in a community setting.^48^

Several patient characteristics predicted mania/hypomania by 12 weeks. In line with previous literature,^8,13,35^ psychosis (for which antipsychotics were an indicator), hospitalizations, and family history of bipolar disorder were associated with 2- to 4-fold increased risks. Additionally, we found the use of antiepileptics to be associated with mania/hypomania. The association may indicate that the patients had shown signs of mood instability shortly after the time of diagnosis.^49^ Antidepressant treatment was unrelated to the risk of mania/hypomania, suggesting that these other characteristics are more relevant when evaluating which patients may have an increased risk of switching from unipolar depression to mania. Our model using administrative information had a moderate predictive ability, suggesting it is possible to identify patients at high risk for mania/hypomania with a prognostic clinical prediction model. The model has potential to be improved in later work.

### Strengths and Limitations

A major strength of this study is its large sample size and recent data covering pediatric psychiatric care in Sweden nationwide. We minimized common biases using the target trial emulation framework and adjusted for several important confounders, such as parental mood disorders. Our results were consistent across 11 robustness checks, including analyses to address potential immortal time bias.

The study also has limitations. Our findings apply primarily to SSRIs. Because we used diagnoses and dispensed medications to measure mania/hypomania, it is possible that some outcomes were misclassified. We did not have information concerning medication dosing and thus cannot rule out that increased dosing contributed to the increased risk in the 52-week follow-up. However, most dosage increases occur early in treatment during titration. Lack of dosing information may also have caused imprecision in the estimated medication exposure periods in per-protocol analyses. In addition, the time frame used for treatment-emergent mania is based on expert consensus. This time frame may be arbitrary since we do not yet know the biological mechanism underlying antidepressant-related adverse reactions. Nevertheless, the data were consistent with no increased risk up to 18 weeks.

## Conclusions

In this cohort study, by 12 weeks of follow-up, we found no significant differences in the risk of mania/hypomania between pediatric patients with unipolar depression treated with antidepressants and those not treated with antidepressants. If antidepressants were to induce mania/hypomania, differences would be expected to emerge by then. A small risk difference was found only in the longer follow-up of 52 weeks, suggesting that the risk increase may be attributable to factors other than the medication.

## Notes

Supplement 1. **eMethods.** Detailed Methods
eReferences
**eTable 1.** Description of Covariates **eTable 2.** Target Trial Emulation Protocol **eFigure 1**. Prescription Time-Distribution Matching to Assign Start of Follow-Up **eTable 3.** Frequencies of Different Antidepressant Medications in the Treatment Group **eTable 4.** Weighted Descriptive Statistics of the Cohort **eFigure 2.** The Estimated Cumulative Incidence of Mania/Hypomania Over 52 Weeks of Follow-Up **eFigure 3.** The Estimated Cumulative Incidence of Mania/Hypomania Over 12 Weeks of Follow-Up **eTable 5.** Sex-Stratified Analyses: Cumulative Incidence and the Relative Risk of Mania/Hypomania in the Treatment and Control Groups at 12 and 52 Weeks of Follow-Up **eTable 6.** Sensitivity Analyses: Non-Weighted Cumulative Incidence and the Relative Risk of Mania/Hypomania in the Treatment and Control Groups at 12 Weeks of Follow-Up

Supplement 2.
Data Sharing Statement
