# Supplementary material for: Antidepressant Use and Risk of Manic Episodes in Children and Adolescents With Unipolar Depression
Source: JAMA Psychiatry. 2023 Sep 27;81(1):25–33. doi: 10.1001/jamapsychiatry.2023.3555 (PMC10534997; doi:10.1001/jamapsychiatry.2023.3555)
Supplement: Supplement 1. — eMethods. Detailed Methods eReferences eTable 1. Description of Covariates eTable 2. Target Trial Emulation Protocol eFigure 1. Prescription Time-Distribution Matching to Assign Start of Follow-Up eTable 3. Frequencies of Different Antidepressant Medications in the Treatment Group eTable 4. Weighted Descriptive Statistics of the Cohort eFigure 2. The Estimated Cumulative Incidence of Mania/Hypomania Over 52 Weeks of Follow-Up eFigure 3. The Estimated Cumulative Incidence of Mania/Hypomania Over 12 Weeks of Follow-Up eTable 5. Sex-Stratified Analyses: Cumulative Incidence and the Relative Risk of Mania/Hypomania in the Treatment and Control Groups at 12 and 52 Weeks of Follow-Up eTable 6. Sensitivity Analyses: Non-Weighted Cumulative Incidence and the Relative Risk of Mania/Hypomania in the Treatment and Control Groups at 12 Weeks of Follow-Up [file jamapsychiatry-e233555-s001.pdf]

Supplementary Online Content

Virtanen S, Lagerberg T, Takami Lageborn C, et al. Antidepressant use and short-term risk of manic episodes in children and adolescents with unipolar depression. *JAMA Psychiatry*. Published online September 27, 2023. doi:10.1001/jamapsychiatry.2023.3555

eMethods. Detailed Methods

eReferences

eTable 1. Description of Covariates

eTable 2. Target Trial Emulation Protocol

eFigure 1. Prescription Time-Distribution Matching to Assign Start of Follow-Up

eTable 3. Frequencies of Different Antidepressant Medications in the Treatment Group

eTable 4. Weighted Descriptive Statistics of the Cohort

eFigure 2. The Estimated Cumulative Incidence of Mania/Hypomania Over 52 Weeks of Follow-Up

eFigure 3. The Estimated Cumulative Incidence of Mania/Hypomania Over 12 Weeks of Follow-Up

eTable 5. Sex-Stratified Analyses: Cumulative Incidence and the Relative Risk of Mania/Hypomania in the Treatment and Control Groups at 12 and 52 Weeks of Follow-Up

eTable 6. Sensitivity Analyses: Non-Weighted Cumulative Incidence and the Relative Risk of Mania/Hypomania in the Treatment and Control Groups at 12 Weeks of Follow-Up

This supplementary material has been provided by the authors to give readers additional information about their work.

© 2023 Virtanen S et al. *JAMA Psychiatry*.

eMethods. Detailed Methods

Per-protocol analysis

To conduct per-protocol analysis where individuals were censored if they switch from on-treatment to off-treatment, or vice versa, we first calculated the estimated treatment periods for individuals who initiated antidepressant medication. A continuous treatment period with an antidepressant was defined based on the assumption that two dispensed prescriptions falling within 120 days of each other belong to the same treatment period. Timeframe of 120 days was selected because oral psychiatric medications are typically not dispensed for more than 90 days at a time in Sweden (so-called 90-day rule). We added 30 days to the 90 days to account for potential treatment non-adherence. Treatment periods were defined independently of the study follow-up. At the last or single dispensation in a treatment period, treatment end date was estimated by adding the study population median number of days between consecutive dispenses to the date of dispensation (40 days in our cohort). This definition is based on prior work [1-2]. Further, we estimated treatment periods for the time-varying psychotropic treatment covariates in the same way as when defining treatment periods for antidepressant medications (i.e., using the 120-day assumption and adding the median number of days).

In per-protocol analyses, individuals initially assigned to the control group were censored on the date they initiated antidepressant medication. Individuals assigned to the treatment group were censored if their estimated treatment period ended before the end of follow-up (at 12/52 weeks). Data were split by week. For each week of follow-up, we assumed that individuals were randomly censored conditional on their baseline and time-varying covariates. The time-varying covariates were indicators for the use of benzodiazepines, antipsychotics, and any other (non-antidepressant) psychotropic drugs over the follow-up (see eTable 1 for variable definitions). All time-varying covariates were updated weekly. We estimated time-varying treatment adherence weights, which accounted for both baseline (covariates specified in the manuscript) and time-varying confounders. We weighted each individual at each week of follow-up by the product of the baseline IPT weights (the same weight also used in the ITT analysis) and the time-varying censoring weights (specified earlier in this section). All weights were stabilized, and truncated at the 99th percentile. We used a discrete-time hazards model (a pooled logistic model) to estimate cumulative incidences and risk ratios for 12 and 52 weeks of follow-up, and calculated 95% confidence intervals for the estimates using non-parametric bootstraps.

We conducted two additional robustness checks for the per-protocol analysis. In the first one, we defined treatment periods assuming that two dispensed prescriptions falling within 60 days of each other belonged to the same treatment period. Treatment end date was estimated in the same manner as in the main approach. In the second robustness check, we estimated treatment periods using the total quantity of medication purchased instead of relying on elapsed time between dispensations. The Prescribed Drug Register includes the size of tablet package (i.e. number of tablets per package) and the number of packages an individual purchased. The duration of treatment period was calculated from the total number of tablets, assuming that the daily dosage was consumed once per day (i.e., 1 pill per day).

Prediction of mania: coding of predictor variables

We investigated which patient characteristics were associated with mania/hypomania in the 12-week of follow-up. For this analysis, we removed individuals with “unknown” as the response category in any of the variables (n=2,197). Some of the predictor variables did not have sufficient number of observations to estimate a separate coefficient. We therefore recoded or combined a subset of the predictor variables to increase statistical power. Family education was recoded as 0=primary school or high school, 1=university level; family income percentile as 0=80th percentile or below, 1=over 80th; number of prior hospitalizations as 0=no hospitalizations, 1=1 or more hospitalizations. Diagnosis for alcohol or drug use disorder were combined into one variable, where having either one of these diagnoses or having both diagnoses were coded as 1, and having neither was coded was 0. In the same manner, we combined 1) conduct disorder and personality disorder diagnosis, 2) poisoning by alcohol and poisoning

© 2023 Virtanen S et al. *JAMA Psychiatry*.

by drugs, 3) the use of benzodiazepines and use other hypnotics/sedatives. The use of opioid medication was dropped from the model, since there were no observations with opioid use which also experienced the outcome.

Sensitivity analysis: cloning-censoring-weighting approach

We conducted cloning-censoring-weighting analysis detailed in Maringe et al. [3]. We used a grace period of 77 days so that the grace period would not be shorter than the primary follow-up of 12 weeks. In this design, follow-up starts at depression diagnosis. At this time, two copies (clones) of each individual are created: one clone is allocated to the treatment group (initiate antidepressant treatment within 77 days of diagnosis), the other clone is allocated to the control group (no initiation of antidepressant treatment within 77 days of diagnosis), forcing the study arms to be identical at baseline. If an individual initiated antidepressants during the grace period, the clone in the control group was censored. If an individual did not initiate antidepressants during the grace period, a clone of the individual remained in each arm for 77 days, after which the clone in the treatment group was censored. Cloning accounts for confounding at baseline, but the artificial censoring is usually informative (i.e., treatment decision is based on characteristics also associated with the outcome), and can therefore introduce selection bias. We addressed this using inverse-probability-of-censoring weighting, with the purpose of up-weighting patients remaining in the risk set so that they represent censored patients, and maintain the comparability of the study arms throughout the grace period. To estimate the weights, we fitted a Cox regression model which was then used to predict the individual probabilities of remaining uncensored at each time of event. The model included covariates specified in the manuscript, and was estimated separately for each arm. The weights are the inverse of these probabilities. We estimated ITT and per-protocol effects. The 120-day gap approach described in the earlier section of eMethods was used to estimate the length of medication treatment periods in the per-protocol analysis. Weighted Kaplan-Meier was used to estimate cumulative incidences, and weighted Cox regression to estimate hazard ratios, in the 12 and 52-week follow-up.

© 2023 Virtanen S et al. *JAMA Psychiatry*.

eReferences

[1] Lagerberg, et al. Selective serotonin reuptake inhibitors and suicidal behaviour: a population-based cohort study. *Neuropsychopharmacology*. 2021:1-7.

[2] Virtanen, et al. Association of selective serotonin re-uptake inhibitor (SSRI) treatment with acute substance misuse outcomes. *Addiction*. 2021;117(1):234-242.

[3] Maringe C, et al. Reflection on modern methods: trial emulation in the presence of immortal-time bias. Assessing the benefit of major surgery for elderly lung cancer patients using observational data. *International Journal of Epidemiology*. 2020;49(5):1719-1729.

© 2023 Virtanen S et al. *JAMA Psychiatry*.

eTable 1. Description of Covariates

Variable	Data source	Timing	ICD or ATC code	Other information
Sex	TPR	Recorded at birth or upon immigration	N/A	
Age	TPR	At the date of diagnosis	N/A	Recorded in years and used as a continuous variable
Parental education level	LISA	On or before the year of child's diagnosis	N/A	If records from several years were available, the information closest to the diagnosis year was selected. If both parental records were available, the education of the highest educated parent was selected.
Family income percentile	LISA	On or before the year of child's diagnosis	N/A	Income distribution was first calculated for the entire Swedish population in each calendar year. Household income of the mother recorded closest to the child's diagnosis year was collected, and the income percentile was calculated based on the entire population estimate.
Diagnosis year	NPR	At the date of diagnosis	N/A	
Source of diagnosis	NPR	At the date of diagnosis	N/A	
Parental bipolar disorder	NPR	Any time between Jan 1997 and the date of child's diagnosis	F30, F31, F250	If either parent had a diagnosis, variable was coded as 1. Coded as 0 if neither parent had a diagnosis. If both parent's information was missing, coded as 2.
Parental depression	NPR	Any time between Jan 1997 and the date of child's diagnosis	F32, F33	If either parent had a diagnosis, variable was coded as 1. Coded as 0 if neither parent had a diagnosis. If both parent's information was missing, coded as 2.
Number of prior hospitalizations	NPR	Any time between Jan 1997 and the start of follow-up	Any F-diagnosis, X40-X49, T36-T50, X60-X84, Y10-Y34	Only diagnoses from hospital/inpatient setting which resulted in an overnight stay were counted.
Anxiety disorder	NPR	Any time between Jan 1997 and the start of follow-up	F4	
Personality disorder	NPR	Any time between Jan 1997 and the start of follow-up	F60, F61	

© 2023 Virtanen S et al. *JAMA Psychiatry*.

ADHD	NPR	Any time between Jan 1997 and the start of follow-up	F90	
Developmental disorder	NPR	Any time between Jan 1997 and the start of follow-up	F7, F80-F98 (excl. F90, F91, F84)	
Autism spectrum disorder	NPR	Any time between Jan 1997 and the start of follow-up	F84	
Conduct disorder	NPR	Any time between Jan 1997 and the start of follow-up	F91	
Alcohol use disorder	NPR	Any time between Jan 1997 and the start of follow-up	F10	
Drug use disorder	NPR	Any time between Jan 1997 and the start of follow-up	F11-F19	
Poisoning by drugs	NPR	Any time between Jan 1997 and the start of follow-up	X40-X44, X46-X49, T36-T50 (excl. T510)	
Alcohol poisoning	NPR	Any time between Jan 1997 and the start of follow-up	X45, T510	
Suicidal behavior	NPR	Any time between Jan 1997 and the start of follow-up	X60-X84, Y10-Y34	
Antipsychotics	PDR	Within 120 days before the start of follow-up	N05A (excl. lithium)	
Hypnotics/sedatives	PDR	Within 120 days before the start of follow-up	N05B (excl. N05BA)	
Benzodiazepines	PDR	Within 120 days before	N05BA	

© 2023 Virtanen S et al. *JAMA Psychiatry*.

		the start of follow-up		
Antiepileptics	PDR	Within 120 days before the start of follow-up	N03A	
Opioids	PDR	Within 120 days before the start of follow-up	N02A	
Stimulants	PDR	Within 120 days before the start of follow-up	N06B	

Note: TPR=Total Population Register; NPR=National Patient Register; PDR=Prescribed Drug Register

© 2023 Virtanen S et al. *JAMA Psychiatry*.

eTable 2. Target Trial Emulation Protocol

Component	Target trial specification	Target trial emulation
**Eligibility criteria**	• Diagnosis of depression (ICD-10: F32-F33) July 1st 2006-Dec 31st 2019, and aged 4-17<br>• No antidepressant (N06A) prescriptions within 365 days before depression diagnosis<br>• No prior diagnosis of mania (F30), bipolar disorder (F31), or any psychosis (F2)<br>• No prior prescriptions for lithium (N05AN), valproate (N03AG01), or carbamazepine (N03AF01) for mood stabilization.	Same as target trial.
**Treatment strategies**	1. Initiation of any antidepressant within 90 days of depression diagnosis<br>2. No initiation of any antidepressant within 90 days of depression diagnosis	Same as target trial<br><br>START OF FOLLOW-UP: Initiators start at time of prescription dispensation; time between depression diagnosis and prescription in initiators is randomly assigned to controls (ensures same distribution of time since diagnosis in initiators and controls)
**Treatment assignment**	Individuals are randomly assigned to a strategy at baseline and will be aware of the strategy to which they have been assigned.	We classified individuals according to the strategy they are compatible with at baseline.<br><br>Participants are assumed to be randomly assigned to treatment at baseline conditional on: sex, age, source of depression diagnosis (inpatient vs. outpatient), year of diagnosis, parental education level, parental income, parental history of mood disorder, previous diagnoses (anxiety, eating disorder, ADHD, drug use disorder, alcohol use disorder, personality disorder, conduct disorder, autism spectrum disorder, developmental disorder, suicide attempt/self-harm, poisoning), medication receipt within last 4 mo.s (antipsychotics, hypnotics and sedatives, benzodiazepines, stimulants, antiepileptics, opioids)
**Outcomes**	Mania (F30), bipolar disorder (F31.0, F31.1, F31.2, F31.6), or the initiation of lithium, valproate, or carbamazepine (for mood stabilization)	Same as for target trial.
**Follow-up**	Starts at randomization and ends at (whichever occurs first): 12 weeks after randomization, death, emigration, hospitalization for psychosis, outcome	Same as for target trial.
**Causal contrasts**	Intention-to-treat (ITT) effect, per-protocol (PP) effect	Observational analog of intention-to-treat and per-protocol

© 2023 Virtanen S et al. *JAMA Psychiatry*.

eFigure 1.

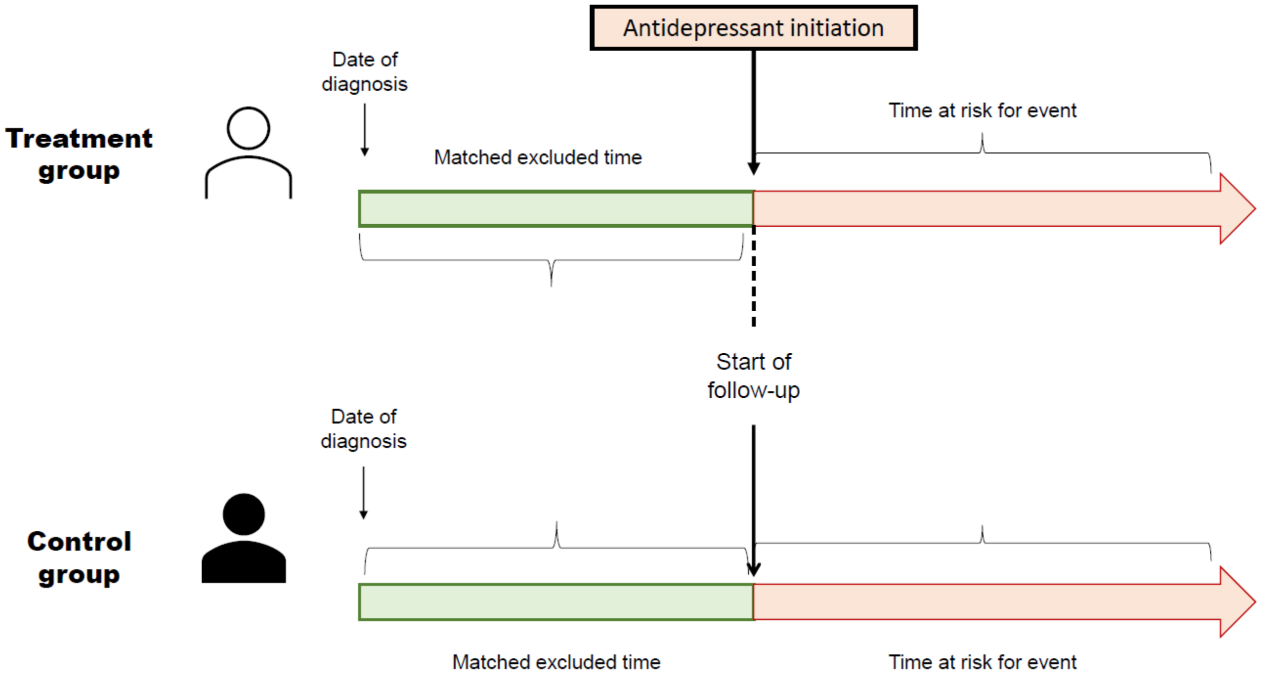

eFigure 1. Prescription Time-distribution Matching to Assign Start of Follow-up

© 2023 Virtanen S et al. *JAMA Psychiatry*.

eTable 3. Frequencies of Different Antidepressant Medications in the Treatment Group

Medication (ATC code)	n	%
Non-selective monoamine reuptake inhibitors		
clomipramine (N06AA04)	N/A	N/A
amitriptyline (N06AA09)	N/A	N/A
nortriptyline (N06AA10)	N/A	N/A
Selective serotonin reuptake inhibitors		
fluoxetine (N06AB03)	11853	48.24
citalopram (N06AB04)	614	2.50
paroxetine (N06AB05)	15	0.06
sertraline (N06AB06)	10305	41.94
fluvoxamine (N06AB08)	13	0.05
escitalopram (N06AB10)	675	2.75
Other antidepressants		
tryptophan (N06AX02)	N/A	N/A
mianserin (N06AX03)	16	0.07
mirtazapine (N06AX11)	893	3.63
bupropion (N06AX12)	85	0.35
venlafaxine (N06AX16)	43	0.17
reboxetine (N06AX18)	N/A	N/A
duloxetine (N06AX21)	36	0.15
agomelatine (N06AX22)	N/A	N/A
vortioxetine (N06AX26)	N/A	N/A

Note: N/A = For these medications, there were too few patients to provide exact numbers without compromising identifiability.

© 2023 Virtanen S et al. *JAMA Psychiatry*.

eTable 4. Weighted Descriptive Statistics of the Cohort

	Control (n=19036.4)	Treatment (n=24436.2)	SMD
Female (%)	12557.1 (66.0)	16150.7 (66.1)	0.003
Age (mean (SD))	14.93 (1.92)	14.95 (1.83)	0.006
Parental education level (%)			0.008
Primary school	777.3 (4.1)	986.1 (4.0)	
High school	8124.3 (42.7)	10399.3 (42.6)	
University	9494.4 (49.9)	12254.1 (50.1)	
Unknown	640.4 (3.4)	796.7 (3.3)	
Family income percentile (%)			0.006
<20	1186.6 (6.2)	1511.7 (6.2)	
20-80	12542.4 (65.9)	16082.4 (65.8)	
>80	4586.5 (24.1)	5933.7 (24.3)	
Unknown	720.9 (3.8)	908.4 (3.7)	
Diagnosis year (mean (SD))	2014.00 (3.70)	2013.99 (3.71)	0.003
Source of diagnosis (%)			0.003
Outpatient	17631.8 (92.6)	22624.9 (92.6)	
Inpatient	1201.6 (6.3)	1554.6 (6.4)	
Unknown	203.0 (1.1)	256.7 (1.1)	
Parental bipolar disorder (%)			0.002
No	17683.0 (92.9)	22713.1 (92.9)	
Yes	811.2 (4.3)	1033.1 (4.2)	
Unknown	542.3 (2.8)	690.0 (2.8)	
Parental depression (%)			0.002
No	15889.6 (83.5)	20393.9 (83.5)	
Yes	2604.1 (13.7)	3352.9 (13.7)	
Unknown	542.7 (2.9)	689.4 (2.8)	
Number of prior hospitalizations (%)			0.009
0	16372.6 (86.0)	20939.9 (85.7)	
1	2016.1 (10.6)	2649.1 (10.8)	
2 or 3	541.2 (2.8)	711.6 (2.9)	
4 or more	106.5 (0.6)	135.6 (0.6)	
Diagnoses (%)			
Anxiety disorder	5605.8 (29.4)	7267.6 (29.7)	0.006
Eating disorder	1426.0 (7.5)	1861.6 (7.6)	0.005
Personality disorder	238.0 (1.3)	305.6 (1.3)	0.000
ADHD	2806.7 (14.7)	3584.8 (14.7)	0.002
Developmental disorder	1693.4 (8.9)	2174.2 (8.9)	0.000
Autism spectrum disorder	1062.0 (5.6)	1382.1 (5.7)	0.003
Conduct disorder	499.8 (2.6)	641.5 (2.6)	0.000
Alcohol use disorder	380.6 (2.0)	493.3 (2.0)	0.001
Drug use disorder	384.8 (2.0)	483.0 (2.0)	0.003
Poisoning by drugs	928.5 (4.9)	1172.9 (4.8)	0.004

© 2023 Virtanen S et al. *JAMA Psychiatry*.

Alcohol poisoning	30.4 (0.2)	38.8 (0.2)	0.000
Suicidal behavior	1192.6 (6.3)	1518.1 (6.2)	0.002
Medications (%)			
Antipsychotics	405.7 (2.1)	531.5 (2.2)	0.003
Hypnotics/sedatives	6919.9 (36.4)	8973.5 (36.7)	0.008
Benzodiazepines	70.9 (0.4)	115.2 (0.5)	0.015
Antiepileptics	211.9 (1.1)	265.2 (1.1)	0.003
Opioids	183.8 (1.0)	236.7 (1.0)	0.000
Stimulants	1711.8 (9.0)	2177.1 (8.9)	0.003

Note: SMD = Absolute standardized mean difference

© 2023 Virtanen S et al. *JAMA Psychiatry*.

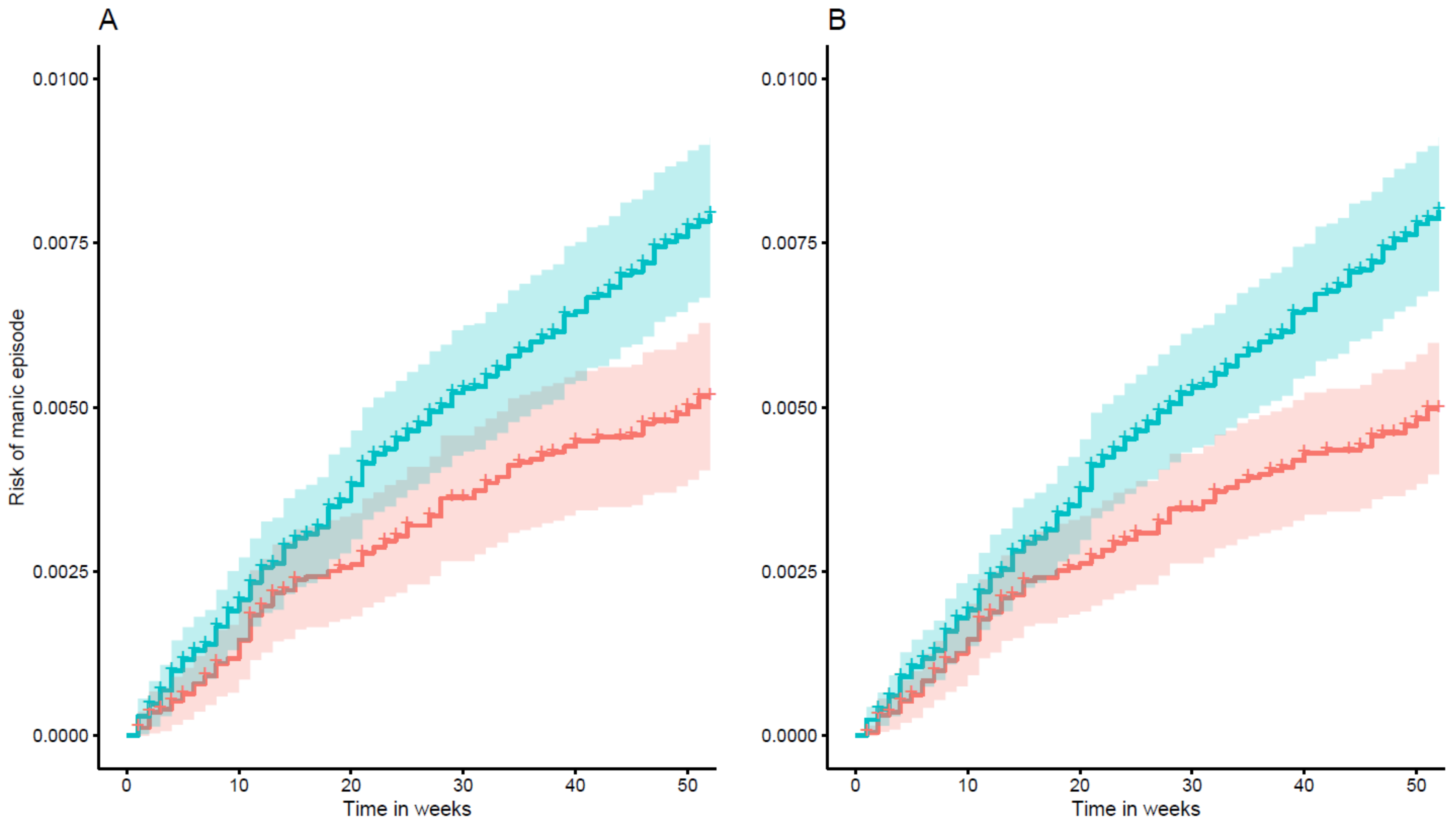

eFigure 2. The Estimated Cumulative Incidence of Mania/Hypomania Over 52 Weeks of Follow-up. Green line represents the treatment group and red line represents the control group. Intention-to-treat estimates. Panel A: weighted data. Panel B: non-weighted data.

© 2023 Virtanen S et al. *JAMA Psychiatry*.

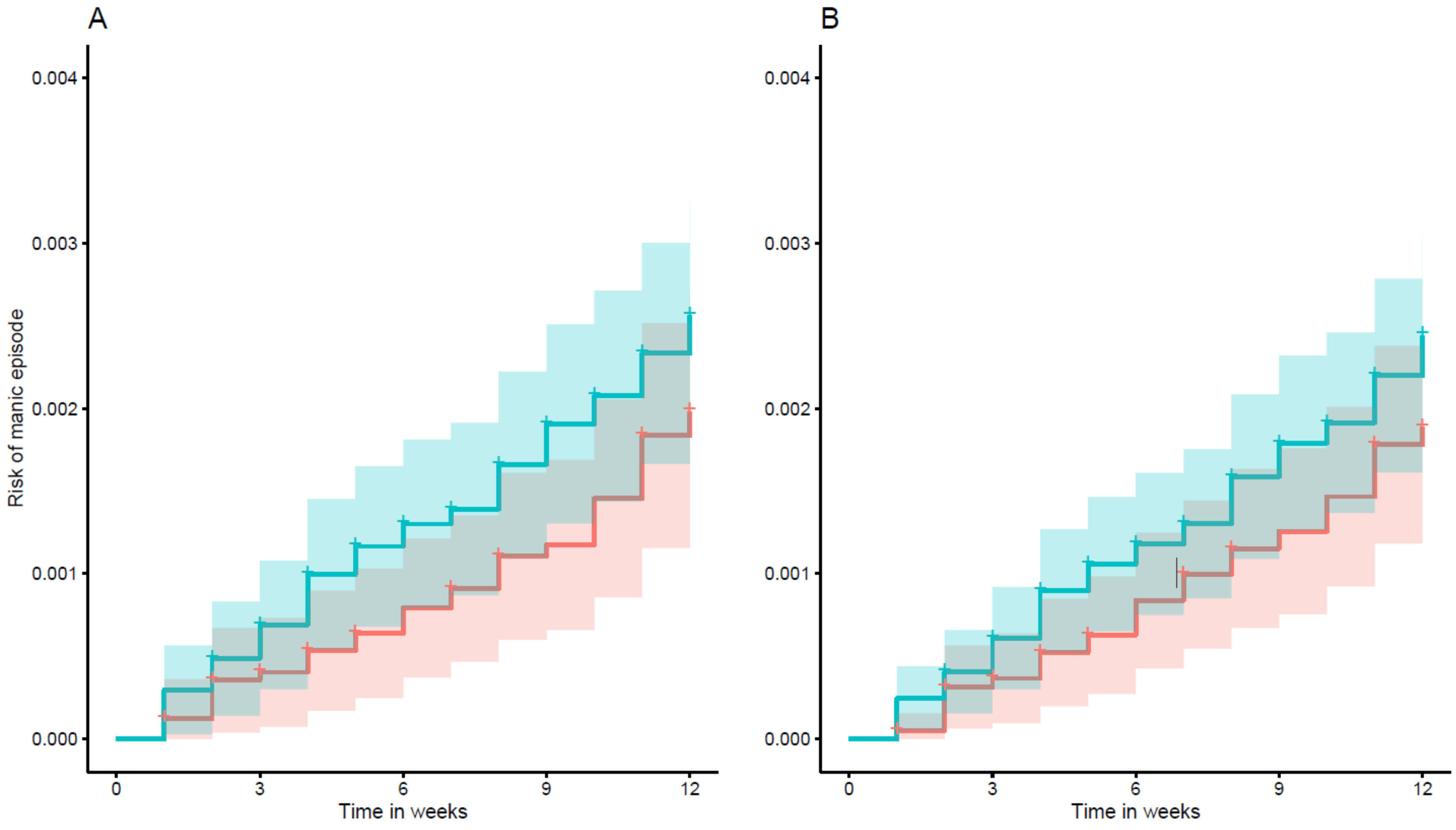

eFigure 3. The Estimated Cumulative Incidence of Mania/Hypomania Over 12 Weeks of Follow-up. Green line represents the treatment group and red line represents the control group. Intention-to-treat estimates. Panel A: weighted data. Panel B: non-weighted data.

© 2023 Virtanen S et al. *JAMA Psychiatry*.

eTable 5. Sex-stratified Analyses: Cumulative Incidence and the Relative Risk of Mania/Hypomania in the Treatment and Control Groups at 12 and 52 Weeks of Follow-up

		N	No. events	Cumulative incidence, %	Risk difference	Hazard Ratio
**Girls**						
12 weeks	Control	12277	23	0.21 (0.14–0.28)		
	Treatment	16608	35	0.21 (0.14–0.29)	0.00 (-0.11–0.12)	1.02 (0.58–1.79)
52 weeks	Control	12277	63	0.54 (0.40–0.67)		
	Treatment	16608	126	0.73 (0.60–0.87)	0.20 (0.00–0.39)	1.37 (0.99–1.89)
**Boys**						
12 weeks	Control	6827	13	0.17 (0.03–0.31)		
	Treatment	7965	25	0.33 (0.19–0.48)	0.16 (-0.01–0.34)	1.96 (0.95–4.05)
52 weeks	Control	6827	32	0.47 (0.25–0.70)		
	Treatment	7965	70	0.90 (0.68–1.13)	0.43 (0.15–0.71)	1.91 (1.23–2.98)

Note: Estimates are from the intention-to-treat analysis

© 2023 Virtanen S et al. *JAMA Psychiatry*.

eTable 6. Sensitivity Analyses: Non-weighted Cumulative Incidence and the Relative Risk of Mania/Hypomania in the Treatment and Control Groups at 12 Weeks of Follow-up

.

	Cumulative incidence, %			
	Control	Treatment	Risk difference	Hazard ratio
Complete cases[a]	0.19 (0.13–0.25)	0.23 (0.17–0.29)	0.04 (-0.05–0.13)	1.20 (0.78–1.84)
Excl. certain medication users[b]	0.16 (0.10–0.22)	0.20 (0.14–0.25)	0.04 (-0.04–0.12)	1.23 (0.78–1.94)
Extended grace period[c]	0.20 (0.14–0.26)	0.25 (0.19–0.31)	0.05 (-0.06–0.15)	1.24 (0.82–1.87)
Restricted outcome definition[d]	0.07 (0.04–0.10)	0.06 (0.03–0.09)	-0.02 (-0.06–0.03)	0.78 (0.37–1.63)
Extended outcome definition[e]	0.20 (0.14–0.26)	0.26 (0.19–0.32)	0.06 (-0.03–0.15)	1.29 (0.86–1.93)
Extended follow-up length[f]	0.25 (0.19–0.32)	0.34 (0.27–0.41)	0.09 (-0.01–0.19)	1.35 (0.94–1.92)
Censoring for any hospitalization[g]	0.17 (0.11–0.23)	0.23 (0.17–0.29)	0.06 (-0.03–0.14)	1.33 (0.86–2.06)
Cloning-censoring-weighting, ITT[h]	0.20 (0.15–0.26)	0.22 (0.18–0.27)	0.02 (-0.05–0.10)	1.28 (0.78–1.62)
Cloning-censoring-weighting, PP[h]	0.20 (0.15–0.26)	0.21 (0.17–0.26)	0.01 (-0.09–0.06)	1.06 (0.73–1.55)
60-day gap treatment periods, PP[i]	0.19 (0.12–0.26)	0.22 (0.16–0.28)	0.03 (-0.06–0.12)	1.16 (0.75–1.79)
1 pill per day treatment periods, PP[j]	0.19 (0.13–0.25)	0.19 (0.14–0.24)	0.00 (-0.07–0.07)	1.00 (0.70–1.45)

Note: PP=per-protocol. Estimates are from the intention-to-treat (ITT) approach unless stated otherwise.

[a] Complete cases model excluded individuals with missing information in any covariate, cohort n=41,480.

[b] Individuals who dispensed antipsychotic or antiepileptic medications within 4 months before follow-up start were excluded, cohort n=42,346.

[c] The grace period was extended to 120 days after diagnosis in defining the treatment group.

[d] We considered outcomes to have occurred only in individuals who had at least two diagnoses, or one diagnosis and one medication dispensation.

[e] Outcome was extended to include dispensations for olanzapine (with indication for “mood stabilizing” purpose).

[f] Follow-up length was extended to from 12 to 18 weeks.

[g] Individuals were censored if they were hospitalized for any mental health reason (excluding the outcome).

[h] Cloning-censoring-weighting approach described in detail in Supplemental eMethods. Follow-up began from the index diagnosis.

[i] When defining antidepressant treatment periods, we assumed that two dispensations falling within 60 days of each other belonged to the same treatment period. See Supplemental eMethods for details of this analysis.

[j] Antidepressant treatment period lengths were estimated from the total quantity of pills dispensed, and assuming that patients take 1 pill per day.

© 2023 Virtanen S et al. *JAMA Psychiatry*.
