# Supplementary material for: Antidepressant Use and Risk of Manic Episodes in Children and Adolescents With Unipolar Depression
Source: JAMA Psychiatry. 2023 Sep 27;81(1):25–33. doi: 10.1001/jamapsychiatry.2023.3555 (PMC10534997; doi:10.1001/jamapsychiatry.2023.3555)
Supplement: Supplement 2. — Data Sharing Statement [file jamapsychiatry-e233555-s002.pdf]

Data Sharing Statement

Virtanen. Antidepressant Use and Risk of Manic Episodes in Children and Adolescents With Unipolar Depression. *JAMA Psychiatry*. Published September 27, 2023.
doi:10.1001/jamapsychiatry.2023.3555

Data
Data available: No

Additional Information
Explanation for why data not available: The data that support the findings of this study are available from Statistics Sweden and The Swedish National Board of Health and Welfare, but restrictions apply to the availability of these data, which were used with ethical permission for the current study and therefore are not publicly available.
